# A Local Adversarial Attack with a Maximum Aggregated Region Sparseness Strategy for 3D Objects

**DOI:** 10.3390/jimaging11010025

**Published:** 2025-01-13

**Authors:** Ling Zhao, Xun Lv, Lili Zhu, Binyan Luo, Hang Cao, Jiahao Cui, Haifeng Li, Jian Peng

**Affiliations:** 1 Department of School of Geosciences and Info-Physics, Central South University, Changsha 410083, China; zhaoling@csu.edu.cn (L.Z.); 225011057@csu.edu.cn (X.L.); binyan.luo@csu.edu.cn (B.L.); 225012156@csu.edu.cn (H.C.); 225001011@csu.edu.cn (J.C.); lihaifeng@csu.edu.cn (H.L.); 2 Department of Hunan Provincial Institute of Land and Resources Planning, Hunan Key Laboratory of Land Resources Evaluation and Utilization, Changsha 410083, China; zllhnghy@163.com; 3 Department of Precision Instrument, Tsinghua University, Beijing 100084, China

**Keywords:** deep learning, adversarial attack, physical attack, 3D object detection, transferability

## Abstract

The increasing reliance on deep neural network-based object detection models in various applications has raised significant security concerns due to their vulnerability to adversarial attacks. In physical 3D environments, existing adversarial attacks that target object detection (3D-AE) face significant challenges. These attacks often require large and dispersed modifications to objects, making them easily noticeable and reducing their effectiveness in real-world scenarios. To maximize the attack effectiveness, large and dispersed attack camouflages are often employed, which makes the camouflages overly conspicuous and reduces their visual stealth. The core issue is how to use minimal and concentrated camouflage to maximize the attack effect. Addressing this, our research focuses on developing more subtle and efficient attack methods that can better evade detection in practical settings. Based on these principles, this paper proposes a local 3D attack method driven by a Maximum Aggregated Region Sparseness (MARS) strategy. In simpler terms, our approach strategically concentrates the attack modifications to specific areas to enhance effectiveness while maintaining stealth. To maximize the aggregation of attack-camouflaged regions, an aggregation regularization term is designed to constrain the mask aggregation matrix based on the face-adjacency relationships. To minimize the attack camouflage regions, a sparseness regularization is designed to make the mask weights tend toward a U-shaped distribution and limit extreme values. Additionally, neural rendering is used to obtain gradient-propagating multi-angle augmented data and suppress the model’s detection to locate universal critical decision regions from multiple angles. These technical strategies ensure that the adversarial modifications remain effective across different viewpoints and conditions. We test the attack effectiveness of different region selection strategies. On the CARLA dataset, the average attack efficiency of attacking the YOLOv3 and v5 series networks reaches 1.724, which represents an improvement of 0.986 (134%) compared to baseline methods. These results demonstrate a significant enhancement in attack performance, highlighting the potential risks to real-world object detection systems. The experimental results demonstrate that our attack method achieves both stealth and aggressiveness from different viewpoints. Furthermore, we explore the transferability of the decision regions. The results indicate that our method can be effectively combined with different texture optimization methods, with the average precision decreasing by 0.488 and 0.662 across different networks, which indicates a strong attack effectiveness.

## 1. Introduction

In recent years, the rapid advancement of deep learning in computer vision has led to the widespread application of convolutional neural networks (CNNs) in 3D object detection and recognition [1,2,3,4]. Recent studies have demonstrated that the recognition capabilities of neural networks are limited and susceptible to minor perturbations in datasets, which results in erroneous predictions [1,5,6]. This phenomenon is called adversarial attacks and involves perturbed samples called adversarial examples. These adversarial examples can deceive neural networks, including various security systems, and pose significant threats to the safety and effectiveness in real-world applications [7,8,9].

Research on adversarial examples has predominantly focused on traditional 2D image adversarial attacks by modifying certain pixels in the digital realm [3,10,11]. In practical applications, it is essential to consider how to translate adversarial attacks from the digital domain to physical-world adversarial disguises. Current methods to generate adversarial patches can be categorized into two types based on the training form: (1) 2D Adversarial Patches: These are generated from 2D images and printed or affixed to real-world objects [12,13,14,15]. Most of these methods are based on gradient optimization techniques such as FGSM [16,17,18], C&W [19], particle swarm optimization (PSO) [20], and reinforcement learning (RL) [21]; (2) 3D Adversarial Examples: These directly modify the shape or texture of 3D objects to achieve adversarial attacks [22,23]. Additionally, 2D image-based adversarial attacks struggle to adapt to the complexities of the real world and are generally less effective in the 3D domain than attacks based on 3D objects. These methods have been applied in various security scenarios such as facial recognition and vehicle identification [24,25,26].

However, three main issues arose from the aforementioned works:

1 For the 2D-3D gradient transfer problem, the adversarial object method is used [27,28,29], which introduces undifferentiable optimization parameters. This makes it challenging to apply gradient-based optimization techniques effectively, leading to suboptimal attack performance and reduced ability to fine-tune adversarial perturbations for enhanced stealthiness.
2 Adapting to multi-angle transformations is difficult: Optical control is used to create an optical effect [30,31,32], so the adversarial attacks are only valid at certain angles. As a result, these attacks lack robustness when the object is viewed from different perspectives, limiting their practicality in dynamic real-world environments where the viewing angles can vary unpredictably.
3 To balance the visual stealth and attack effectiveness, the texture or shape of the target is modified [33,34,35], which results in camouflages that may be too large to be hidden by artificial vision or may be small but have less attack effectiveness. This trade-off often forces a compromise where either the camouflage becomes easily noticeable, thereby compromising stealth, or it remains inconspicuous but fails to achieve the desired level of attack effectiveness, undermining the overall goal of the adversarial attack.

These limitations highlight the need for more sophisticated attack strategies that can maintain high levels of stealth while ensuring robust and effective adversarial performance across various real-world conditions. Thus, a new method that can comprehensively handle these challenges is needed.

In this paper, we propose a local adversarial attack with a Maximum Aggregated Region Sparseness (MARS) strategy. To address the issues of 3D-2D gradient propagation in the physical world and complex environmental factors, we employ a neural rendering method to obtain differentiable multi-angle augmented data. To balance the attack efficiency and visual stealth, we maximize the aggregation and minimize the attack camouflage under the guidance of object detection. To maximize the aggregation of attack camouflage regions, an aggregation regularization term is designed to constrain the mask aggregation matrix based on the face-adjacency relationships. To minimize the attack camouflage regions, sparseness regularization is introduced, which makes the mask weights tend toward a U-shaped distribution and limits extreme values. By suppressing model detection to locate universal critical decision regions from multiple angles, texture modifications in these regions achieve universal local camouflage. The main contributions of this paper are as follows:

1 We propose a local 3D attack framework driven by the Maximum Aggregated Region Sparseness (MARS) strategy, which ensures visual stealth while achieving efficient attacks.
2 We design an optimization regularization specifically for 3D mesh faces, including aggregation regularization to maximize the aggregation of attack camouflage regions and sparseness regularization to minimize the attack camouflage regions. This approach effectively adapts to the characteristics of deep neural networks and identifies critical decision regions on 3D objects.
3 Compared with the baseline methods, the MARS attack strategy achieves an average attack efficiency (AE) of 1.724, i.e., an improvement of 0.986 (134%). Additionally, MARS effectively attacks vehicles from different viewpoints, so it is robust to angle variations. Transferability experiments indicate that MARS exhibits strong attack performance in black-box environments.

Our paper is organized as follows: Section 2 illustrates the related work, and Section 3 describes the details of our proposed method. The experiments and conclusions are presented in Section 4 and Section 5.

## 2. Related Work

In this section, we introduce the recent exploration of physical adversarial examples and rendering.

### 2.1. Physical Adversarial Attack

Recently, the rapid advancement of deep learning in the domain of computer vision has significantly transformed the process of automated understanding of realistic images [36,37,38,39,40], and it has found widespread application across diverse professional domains [41,42,43,44,45]. Nevertheless, research into adversarial attacks on realistic images for deep learning is also progressing [46,47]. Example adversarial attack algorithms can be divided into digital attacks and physical attacks based on their implementation domains. In physical scenarios, numerous factors such as lighting and camera parameters affect the adversarial attack and make its implementation more complex. Currently, physical adversarial attacks can be classified into three categories based on the attack method: adversarial objects, optical adversarial attacks, and attribute modifications of the target object.

**1. Adversarial Objects:** This approach involves placing objects with specific shapes and textures on or near the target object. For example, Tsai et al. [48] implemented robust adversarial objects in both digital and physical worlds using point cloud data for PointNet++. Lee et al. [49] proposed the first practical method to generate 3D adversarial point clouds. Liu et al. [50] extended this field with additional attack methods and metrics. Kotuliak et al. [51] used GANs to create unrestricted false-positive adversarial examples. Cao et al. [52] introduced LIDAR-ADV, which generates adversarial objects that evade LIDAR-based detection systems under various conditions, to reveal potential vulnerabilities in autonomous driving detection systems. Although these methods are easy to implement in the physical world, they often lack differentiable optimization and have poor attack effectiveness. They can also be easily detected, removed, or neutralized by simple defense mechanisms such as random sampling and outlier removal, as described by Liu et al [50]. In contrast, our method enhances both the effectiveness and stealthiness of attacks by maximizing the sparsity of aggregated regions. This allows for stronger attacks within smaller areas, overcoming the limitations of existing approaches.

**2. Optical Adversarial Attacks:** These attacks involve manipulating light using optical instruments and can be categorized based on the stage of the imaging process. Light capture manipulation: Hu et al. [53] proposed AdvZL, which successfully attacks autonomous driving tasks using zoom lenses. Translucent patches [54] use transparent stickers to cover the camera lens and hide the target. Light source manipulation: Li et al. [55] used adversarial illumination to attack structured-light-based 3D face recognition, whereas AdvSL [56] used spotlights for flexible attacks in complex environments. Shadow creation: Zhong et al. [57] created shadows on traffic signs to produce naturalistic and stealthy optical adversarial examples. Wang et al. [58] proposed Reflective Light Attack (RFLA), which is effective in bright environments. These methods struggle to adapt to the complexities of the physical world and are only effective under specific conditions. In contrast, our approach leverages neural rendering techniques to enable gradient propagation across multiple angles, ensuring that attacks remain effective and stealthy from various viewpoints. This significantly enhances adaptability in complex environments.

**3. Attribute Modifications of the Target Object:** This category includes modifying the shape of the target [29], which is challenging to reproduce in the physical world, and modifying the texture of the target, which is performed in this paper. Texture modification is differentiable in the digital world and easy to reproduce in the physical world. Numerous studies have focused on physical adversarial attacks with different texture coverages. Full object coverage: Pautov et al. [18] projected adversarial patterns onto a mesh using a grid generator to simulate nonlinear transformations for facial recognition. Komkov et al. [16] used a spatial transformation layer (STL) to project perturbations onto a hat. Partial object coverage: Wei et al. [59] optimized the position and rotation angle of cartoon stickers to evade facial recognition systems, which made this method easier to implement and more threatening. DAS [60] combines meaningful and meaningless patches using smiley outlines (to attract attention) and unrecognizable patterns (to deceive DNNs). However, these methods often face a trade-off between coverage and attack effectiveness, making it difficult to achieve strong attacks while maintaining high stealthiness. In contrast, our proposed MARS strategy effectively achieves powerful attacks within limited regions by maximizing the sparsity of aggregated areas, balancing both stealth and attack strength.

In summary, due to safety and cost constraints, the existing adversarial camouflage methods often require large areas to cover the surface of an object, which results in visually obvious full object coverage attacks or inefficient partial attacks that only work on a limited number of angles. In contrast, our method employs the Maximum Aggregated Region Sparseness strategy to achieve more efficient attacks within smaller regions while maintaining effectiveness across multiple viewpoints. This significantly enhances both the practicality and stealthiness of the attacks. Therefore, this paper aims to find a method that balances the attack area and attack effectiveness.

### 2.2. Rendering in Adversarial Examples

As interest in physical adversarial attacks has grown, rendering in adversarial examples has also garnered the attention of researchers. The goal of rendering is to simulate 2D images of 3D objects under various environmental conditions. Traditional rendering [61,62] involves providing a series of inputs such as geometry, lighting, materials, and camera positions to rasterization or ray-tracing renderers. These renderers output images after a series of pipeline operations. The main challenge with these methods is the propagation of gradient information before and after rendering. To ensure that the rendering process is differentiable, the concept of neural rendering has been introduced. A neural render can be considered a universal function approximator that uses neural networks to transform various parameters into output images. Existing neural rendering methods primarily use differentiable renderers [63,64,65] to simulate rendering processes. For example, Zhang et al. [14] utilized the expectation over transformation (EOT) principle that Athalye et al. [66] proposed to train neural networks for approximate differentiation and simulate the imaging process. STA [67] analyzed the vulnerabilities of Siamese networks in visual tracking and proposed a differentiable rendering pipeline to generate perturbed texture maps for 3D objects, which successfully reduced the tracking accuracy and caused a tracker drift. Complex mapping-based neural rendering, which was used in this paper, facilitates gradient-based optimization. For example, the Neural Mesh Renderer [68] in MeshAdv [69] quickly integrates rendering into neural networks. Yang et al. [70] experimented with various renderers [71,72,73,74] and analyzed their transferability in adversarial attacks to better understand adversarial attacks on 3D objects in the real world.

Currently, most renderers struggle to efficiently propagate gradients from 2D pixels to 3D mesh faces in the model recognition results. This limitation severely affects the generation of adversarial attack models based on 3D targets. In contrast, our proposed rendering method optimizes the gradient transfer process, significantly enhancing the efficiency and effectiveness of generating adversarial attack models for 3D targets. This provides robust support for efficient physical adversarial attacks. Therefore, this paper presents an efficient 3D-2D rendering method that facilitates fast gradient transfer.

## 3. Method

In this section, the details of the proposed method are presented, including the regularization for attack patch aggregation, regularization for attack camouflage minimization, and propagation and backpropagation between 3D objects and 2D images. The pseudocode for our algorithm is also demonstrated.

### 3.1. Framework

**Figure 1:**
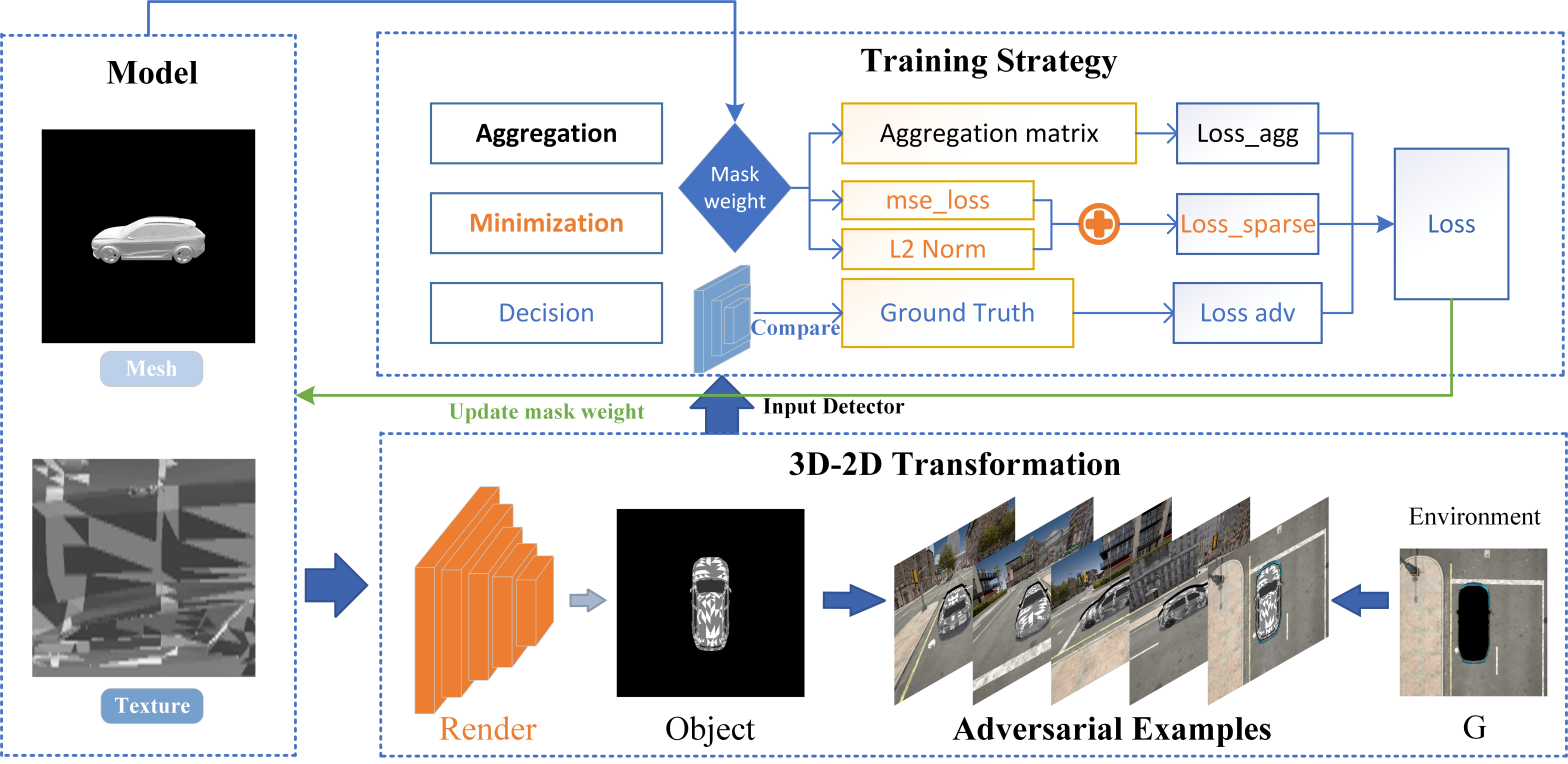
A local adversarial attack with a Maximum Aggregated Region Sparseness strategy for 3D objects.

The Maximum Aggregated Region Sparseness (MARS) strategy is designed to enhance the stealth and effectiveness of local adversarial attacks on 3D objects. MARS operates by identifying and modifying critical decision regions on the target surface in a way that minimizes the visibility of perturbations while maximizing their impact on the detection model. The core of a local adversarial attack with the Maximum Aggregated Region Sparseness (MARS) strategy on 3D objects is to identify critical decision regions on the target surface. Based on the imaging principles of optical remote sensing and to address the optimization challenges in these critical regions, our framework is divided into the following components: (1) To maximize the aggregation of attack camouflage regions, an aggregation regularization term is designed to constrain the mask aggregation matrix based on face-adjacency relationships. (2) To minimize the attack camouflage regions, sparseness regularization is introduced, which drives the mask weights toward a U-shaped distribution and limits extreme values. (3) To facilitate the gradient propagation between 3D objects and 2D images, the camouflage is set as a fixed perturbation to obscure the original texture information. A neural rendering algorithm is used to introduce mask weights that control the proportion of camouflage to the original texture and ensure the differentiability of the optimization parameters. To achieve efficient attacks from multiple angles and distances, the renderer outputs many target images with varied camera parameters $O=R(M,T;\theta_{c})$, which are combined with the real background *G* to produce realistic images I=O+G. By minimizing the target confidence loss under the aggregation and sparseness regularization constraints, the framework learns the shape and position of patches on the target surface. This process identifies the critical decision regions on the target surface and enables effective attacks by altering only small portions of these regions. The framework of MARS is shown in Figure 1.

**Overview of MARS:** The MARS framework operates by balancing two key constraints: aggregation and sparsity. The aggregation constraint ensures that the adversarial perturbations are concentrated in specific, critical regions of the object, enhancing the attack’s effectiveness. The sparsity constraint limits the number of regions being modified, maintaining the stealthiness of the attack by preventing large, noticeable changes. Additionally, the use of a U-shaped weight distribution encourages the mask weights to adopt extreme values (close to 0 or 1), which simplifies the perturbation pattern and makes it less detectable. The process diagrams for each part are shown in Figure 1.

### 3.2. Generating Adversarial Camouflage Regions

#### 3.2.1. Regularization for Attack Camouflage Aggregation

Observations during training reveal that without special constraints, the resulting decision regions may become fragmented, which leads to poor attack performance after the texture iteration. Considering the recognition patterns of deep neural networks, the obtained decision regions must be as aggregated as possible. This aggregation facilitates the creation of complete patterns in subsequent iterations and enables successful attacks by modifying only small areas.

**Figure 2:**
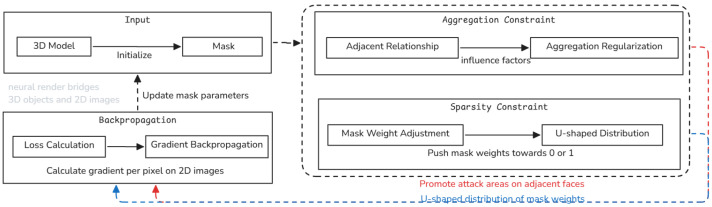
Overview of the Maximum Aggregated Region Sparseness strategy.

To define the degree of aggregation of decision regions, we calculated the sum of mask weights for each face and its adjacent faces as an influencing factor for that face. This parameter is incorporated into the loss function. The iterative computation involves multiple adjacency results: in each iteration, the outermost faces are removed to obtain the weight values of the inner faces, and the process continues until the weight of the central-most face is determined; then, the iteration is stopped. The weight calculation of each face in every iteration considers directly adjacent faces and the neighbors of those adjacent faces (Figure 2).

There are three known adjacency situations for face patches: independent (0 adjacent faces), 1 adjacent face, and 2 adjacent faces. Therefore, the adjacency faces of edge patches must be ≥2. The list is initialized as an exterior face list, where each face is assigned a weight (face_weight) as a transparency value to control the surface texture. In each iteration, the core of each face in the list is calculated as core=face_if·face_weight. After the calculation, the new core value replaces the original weight value. At the end of a single iteration, faces with fewer than 2 adjacent faces are removed from the list. The iteration is repeated until the list length is ≤1. The final aggregation loss is calculated as follows:

(1) $$L_{agg}=sum\left[list_{exterior\_face}\left(core\right)\right]$$

**Figure 3:**
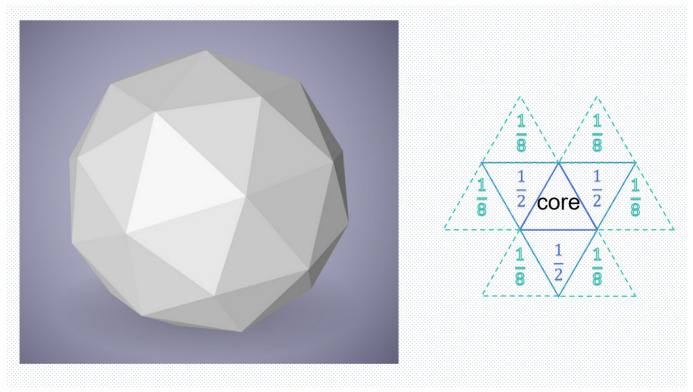
Aggregation matrix: influence factor calculation based on face–core distance.

The aggregation matrix, as shown in Figure 3, indicates that the influence factors of each face on the core can be 0–1. Since the aggregation results replace the original weights during our calculations, we must ensure that the sum of the influence factors of adjacent faces exceeds 1. Based on experiments and empirical data, we scientifically set the influence factors using the distance of each face from the core (i.e., the number of edges apart), which is calculated as follows:

(2) $$face_{if}=Max_{N}/\left(2^{distance}\cdot num\right)$$

where $Max_{N}$ is the maximum number of adjacencies possible in this form to balance the size of the influence factors of adjacent faces; distance is the number of edges from the core; and num is the number of faces in different adjacency forms, such as three faces in the first layer of edge-to-edge adjacency and six faces in the second layer of edge-to-edge-to-edge adjacency.

The aggregation regularization encourages the adversarial patches to form contiguous regions rather than scattered points. By considering the adjacency relationships, the model ensures that modifications are concentrated in clusters, which makes the perturbations more effective and less detectable.

Since all parameters in this regularization are differentiable for each face, the gradient propagation in subsequent stages is not affected, which ensures that this optimization method is feasible. The goal of this part is to minimize $L_{agg}$, aggregate the important faces as much as possible in the optimization results, and consequently reduce the number of optimized regions.

#### 3.2.2. Regularization for the Attack Camouflage Minimization

The purpose of this regularization is to ensure that the attack camouflage area remains within a certain range during training to accelerate the optimization process. To achieve a balanced distribution of important regions, simply considering other losses may lead to continuously monotonous gradients, which are insufficient to balance the area and adversarial effectiveness. Therefore, the attack camouflage minimization must be incorporated into the training process.

**Figure 4:**
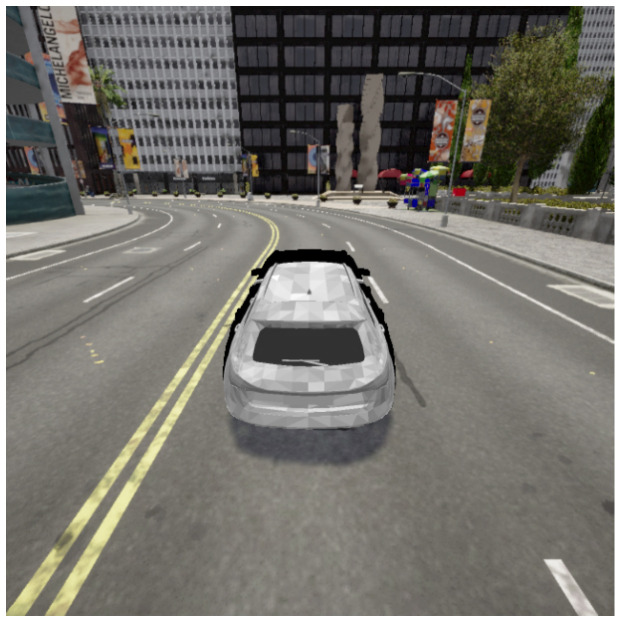
Adversarial examples without regularization for sparsity.

We observe that during training, many faces with uniformly distributed mask weights occupy large areas but hardly contribute to the attack effectiveness. We refer to this phenomenon as “mask uniform distribution.” These masks achieve attacks through a specific set of camouflages with varying transparencies, which contradicts our intention of using fixed perturbations and fails to obscure texture features. The identified regions lack decisiveness and transferability, as shown in Figure 4.

To prevent the adversarial patches from being too spread out and noticeable, we introduce a sparsity constraint. This encourages the mask weights to adopt a U-shaped distribution, where most weights are pushed towards 0 or 1. Such a distribution ensures that only specific regions are heavily modified (weights near 1), while the rest remain largely unchanged (weights near 0), enhancing stealthiness and ensuring that perturbations are concentrated where they are most effective.

To address this issue, we use the Mean Square Error (MSE) loss to constrain the weight distribution and encourage the weights to trend toward 0 or 1 during training. This step prevents certain combinations of patterns from impacting the detection results. The MSE loss is calculated as follows:

(3) $$L_{mse}\left(H\left(M\right),I\right)=\sum_{i=1}^{m}{\left(H\left(M_{i}\right)-1\right)}^{2}/m$$

where *I* is an all-one matrix, *M* is the mask weight matrix, *m* is the number of faces, and H(M) is the matrix form of the array for calculation.

The attack camouflage minimization is divided into two parts. The first part restricts the mask weight of each face to approaching 0 or 1. The second part limits the number of faces with weights approaching 1 to select important regions. The mask weight defines the minimization and represents the opacity level of the fixed perturbation on each face (0 indicates no perturbation, and 1 indicates high visibility of the perturbation). The attack camouflage region minimization is defined as the sum of the power functions of the face weights. To maintain consistency with the MSE loss, the L2 norm is used for sparsity constraints. The sparsity coefficient loss is calculated as follows:

(4) $$L_{sparse}=L_{mse}\left(H\left(M\right),I\right)+{\alpha ||M||}^{2}$$

#### 3.2.3. Total Loss

The total loss consists of three parts: $L_{agg}$ is the aggregation of the attack regions, $L_{sparse}$ is the area of the attack regions, and $L_{adv}$ is the adversarial attack effectiveness:

$L_{agg}$ Loss: To aggregate the adversarial camouflage regions and enable the MARS algorithm to generate more comprehensive regions and enhance the local attack performance, $L_{agg}$ is included in the total loss, as calculated by Equation (1).

$L_{sparse}$ Loss: To minimize the adversarial camouflage regions and enable the MARS algorithm to produce smaller regions with more natural visual effects, $L_{sparse}$ is included in the total loss, as calculated by Equation (4).

$L_{adv}$ Loss: To ensure the decision of adversarial camouflage regions, $L_{adv}$ is included in the total loss; its primary function is to reduce the accuracy of the detection model after the adversarial attacks to achieve the desired adversarial effectiveness. The $L_{adv}$ loss consists of three parts: the bounding box regression loss measures the difference between the detection box and the original ground truth box; the class loss $L_{cls}$ calculates the difference between classification and the true class; and the object loss $L_{obj}$ reflects the object confidence. The Binary Cross-Entropy (BCE) loss is used here, and the confidence label *L* and predicted confidence *P* are used to compute the overall confidence loss. To achieve efficient target attacks, these losses are scaled down by specific hyperparameter ratios during the training process. Additionally, to better control reproducibility in the physical world, we incorporate a smoothness constraint during the texture modification process to regulate color transitions. The smooth loss $L_{smooth}$ is calculated via Equation (8). $L_{adv}$ is calculated via Equation (9):

(5) IoU=(A∩B)/(A∪B)

(6) $$L_{bbox}=\sum_{scale=1}^{3}IoU_{scale}$$

(7) $$L_{cls}=\sum_{cls=1}^{n}BCE\left(p_{cls},\hat{p_{cls}}\right)$$

(8) $$L_{smooth}=\sum_{i,j}{\left(x_{i,j}-x_{i+1,j}\right)}^{2}+{\left(x_{i,j}-x_{i,j+1}\right)}^{2}$$

(9) $$L_{adv}=L_{bbox}+L_{obj}+L_{cls}+L_{smooth}$$

In summary, the total loss function is designed as follows: Hyperparameters α and β control the gradual convergence of the perturbation patches to the desired number during the training process. Simultaneously, parameter γ must occupy a certain proportion to ensure the aggressiveness of the results. An ablation study on the hyperparameter selection is subsequently conducted.

(10) $$L=\alpha \cdot L_{agg}+\beta \cdot L_{sparse}+\gamma \cdot L_{adv}$$

### 3.3. Three-Dimensional–Two-Dimensional Transformation

This section consists of two parts: (1) forward propagation, where 3D objects are transformed into 2D images, and (2) backward propagation, where the loss and gradients from the 2D images are returned to the 3D objects.

**Forward propagation.** The process of generating images from the 3D world is known as rendering, which serves as the boundary between the 3D world and 2D images and is crucial in computer vision. In this method, we use Neural Render, which uses polygon meshes as the 3D format and is represented by a few parameters to denote the 3D shape. This step enables the transformation of a 3D target into many 2D images that contain environmental information, which can be input into the detector for detection and gradient backpropagation.

The render pipeline converts vertices {V_O_I} in the object space to vertices {V_S_I} in the screen space. The rasterization process samples the vertices and faces to generate images and renders each face with its own $s_{t}\cdot s_{t}\cdot s_{t}$ texture map. Barycentric coordinates determine the corresponding texture space coordinates for a position *p* on the triangle {v1,v2,v3}, and bilinear interpolation samples from the texture image to generate the final image.

(11) $$T=a_{p}\cdot mask\_weight+o_{t}\cdot \left(1-mask\_weight\right)$$

This process specifically involves converting the 3D model (M,T) into 2D images *O* that contain adversarial camouflage information. Here, *M* is the model mesh, and *T* is the model texture, which includes both texture and mask. The texture is calculated via Equation (11), where $a_{p}$ denotes the adversarial perturbation and $o_{t}$ signifies the original texture. Environmental image *B* is obtained by segmenting the original image to exclude target information. Final image *I* is created by compositing adversarial image *O* with environmental image *B*.

**Backward propagation.** Neural Render treats the rasterization process as one that can propagate the gradients backward to establish a deep relationship between the 3D target and 2D image, which enables optimization. A neural rendering network is trained here, introducing a suitable approximate gradient for neural network rendering and propagating the gradients to the texture, lighting, camera, and object shape.

To ensure effective gradient backpropagation, we employ linear interpolation to smooth out abrupt changes in color, which helps maintain meaningful gradient information. The U-shaped weight distribution facilitates this by pushing mask weights to their extremes, ensuring that gradients are either fully propagated or completely suppressed, avoiding intermediate values that could dilute the effectiveness of the adversarial perturbations.

Gradient backpropagation is challenging because sudden color changes can lead to zero gradients and prevent backpropagation. Therefore, linear interpolation is used to replace gradual changes among the pixels, as shown in Equation (12). The gradient at $x_{0}\left(x_{0}\in \left[a,b\right]\right)$ is provided by Equation (13), which distinguishes between two cases: When the target pixel $P_{j}$ is inside or outside the face, the partial derivative is defined as zero, and the internal face color is used for the forward propagation to avoid color leakage. If $P_{j}$ is outside the face, the linear interpolation alters the color coefficient, so the derivatives on the left and right sides of $x_{0}$ are calculated, and their sum yields the gradient at $x_{0}$. The specific formulas are provided in Equations (14)–(16).

(12) $$\frac{\partial I_{j}\left(x_{i}\right)}{\partial x_{i}}\;\mathrm{becomes}\;\frac{\delta_{j}^{I}}{\delta_{i}^{x}}$$

(13) $$\frac{\partial I_{j}\left(x_{i}\right)}{\partial x_{i}}{|}_{x_{i}=x_{0}}=\left\{\begin{array}{ccc} \frac{\delta_{j}^{I}}{\delta_{i}^{x}} & , & \delta_{j}^{P}\delta_{j}^{I}<0\\ 0 & , & \delta_{j}^{P}\delta_{j}^{I}\leq 0 \end{array}\right.$$

(14) $$\frac{\partial I_{j}\left(x_{i}\right)}{\partial x_{i}}{|}_{x_{i}=x_{0}}=\frac{\partial I_{j}\left(x_{i}\right)}{\partial x_{i}}{{|}_{x_{i}=x_{0}}^{a}+\frac{\partial I_{j}\left(x_{i}\right)}{\partial x_{i}}|}_{x_{i}=x_{0}}^{b}$$

(15) $$\frac{\partial I_{j}\left(x_{i}\right)}{\partial x_{i}}{|}_{x_{i}=x_{0}}^{a}=\left\{\begin{array}{ccc} \frac{\delta_{j}^{\left(I^{a}\right)}}{\delta_{x}^{a}} & , & \delta_{j}^{P}\delta_{j}^{a}<0\\ 0 & , & \delta_{j}^{P}\delta_{j}^{a}\leq 0 \end{array}\right.$$

(16) $$\frac{\partial I_{j}\left(x_{i}\right)}{\partial x_{i}}{|}_{x_{i}=x_{0}}^{b}=\left\{\begin{array}{ccc} \frac{\delta_{j}^{\left(I^{b}\right)}}{\delta_{x}^{b}} & , & \delta_{j}^{P}\delta_{j}^{b}<0\\ 0 & , & \delta_{j}^{P}\delta_{j}^{b}\leq 0 \end{array}\right.$$

During the backpropagation, if multiple faces overlap, the intersection points are checked to determine whether they are rendered. If they do not overlap with the face, the gradient is not calculated.

### 3.4. Pseudocode for MARS

This section describes the Maximum Aggregated Region Sparseness (MARS) strategy-driven local 3D attack framework, which explains the optimization process of a local adversarial attack with the MARS strategy on 3D objects. To balance the visual stealth and attack effectiveness, we design the MARS algorithm to identify critical decision regions on the target. Using the characteristics of these critical regions and combining them with the network detection results, we design an effective loss function. The gradients of this loss function are backpropagated to guide the parameter updates on the surface of the target object, which models the identification of critical decision regions as an optimization problem. Finally, texture modifications are performed in these regions to achieve local adversarial camouflage with high attack effectiveness. Algorithm 1 outlines the local adversarial attack optimization algorithm based on local decision regions, where *C* represents the texture optimization method in the regions.
**Algorithm 1** MARS**Input:** 3D model (M,T), camera parameter $\theta_{c}$, ground truth label $y_{gt}$**Output:** Aggregated Regions mask $M_{adv}^{*}$1:Initialize $M_{adv}^{*}$ with {0}, adversarial perturbation $A_{adv}$ with background color2:**for** the max iteration **do**3:   **for** the max batch size **do**4:     update mask5:     $I=R(M,T,\theta_{c})$6:     $T_{adv}^{*}=T\cdot \left(1-M_{adv}^{*}\right)+A_{adv}\cdot M_{adv}^{*}$7:     $I_{adv}=R\left(M,T_{adv}^{*},\theta_{c}\right)$8:     $y=D(I_{adv})$9:     calculate L by Equation (10)10:     update $M_{adv}^{*}$ with gradient backpropagation11:   **end for**12:**end for**13:$M_{adv}^{*}=M\left(M,T\right)$14:$T_{adv}^{*}=C\left(M,T,M_{adv}^{*}\right)$

## 4. Experiments and Results

In this section, we illustrate the details of our experimental setup and present the experimental results.

### 4.1. Experimental Setup

**Training Settings**: All models were trained for 20 epochs using the Adam optimizer with an initial learning rate of 0.02. The learning rate was scheduled to decay by a factor of 0.1 every five epochs. A batch size of 2 was used for training, and weight decay was set to 0.0005 to prevent overfitting. These hyperparameters were selected based on preliminary experiments to balance training efficiency and model performance.

**Figure 5:**
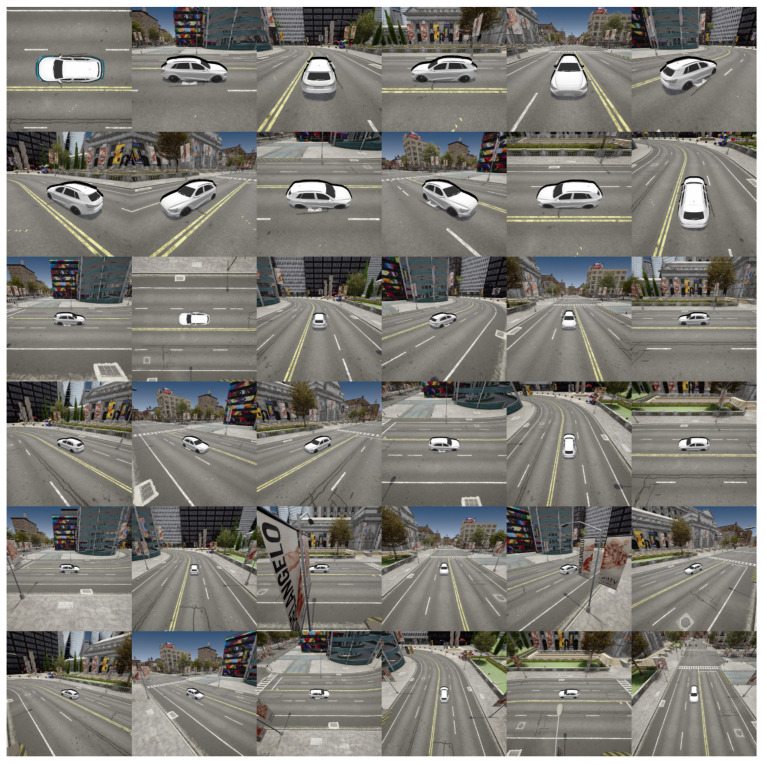
Overview of the dataset which contains a variety of simulated images from the Carla simulation, captured under different perspectives, distances, and environmental conditions.

**Dataset**: Unfortunately, in the field of autonomous vehicle detection, there is no comprehensive and open training dataset suitable for 3D adversarial attacks. Therefore, we chose a rendering-based generative dataset, which offers rich scenes and differentiable rendering. The Carla dataset is the basic dataset in this field, and to be consistent with the domain, the experimental dataset used in this study is the Carla dataset [75]. Therefore, we use the Carla simulator, which is an open simulation platform to simulate the process of a vehicle driving in the city, to generate remote simulation pictures under different perspectives, distances, and environments during the driving process. In total, 15,000 pictures are generated, which are exported from the Carla simulator as the Carla dataset standard, as shown in Figure 5.

**Metric**: To measure the effectiveness of the adversarial attack, we use two metrics: average precision (AP) and a custom metric called attack efficiency (AE). In this experiment, the AP is defined as the average precision for the class “car,” which is calculated using Equation (17). The attack efficiency (AE) is calculated using Equation (18):

(17) $$AP=\int_{0}^{1}p\left(r\right)\;dr$$

(18) $$AE=\frac{\delta_{AP}}{p_{face}}$$

where p(r) is the smoothed precision-recall curve, $\delta_{AP}$ is the difference in average precision before and after the attack, and $p_{face}$ is the proportion of faces modified during the attack. A reduction in AP indicates a decrease in the model’s ability to correctly detect and classify objects, thereby demonstrating the effectiveness of the adversarial attack. However, it is crucial to balance this reduction to avoid overly degrading the model’s performance, which could render the attack impractical or easily noticeable.

**Experimental Schema**: First, we train six detectors from the YOLOv3 and YOLOv5 series on the Carla dataset. We use three selection strategies (manual expert selection, random selection, and MARS) to identify different local regions. Full-body attacks are used as the baseline control group to compare and analyze the attack effects on these regions. The training was conducted under identical conditions for all models to ensure a fair comparison.

The experiments are divided into three parts:

1 Attack efficiency: the main objective is to demonstrate the superiority of MARS;
2 Transferability: this part assesses the transferability of the model by comparing the impacts of different texture optimization methods on the attack performance;
3 Parameter sensitivity analysis: this section presents an investigation of the significance and sensitivity of core parameters through variations and ablation experiments.

### 4.2. Local Adversarial Attacks with Different Region Selection Strategies

**Figure 6:**
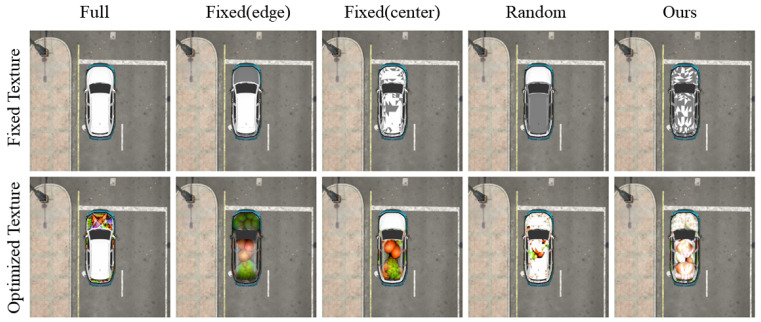
Examples of different region selection strategies.

**Figure 7:**
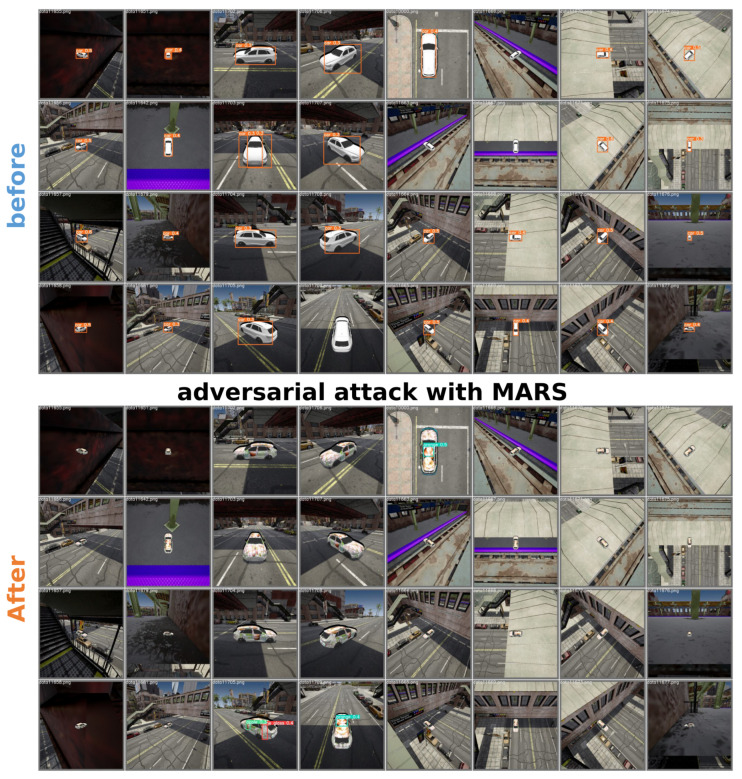
Adversarial examples generated from different angles based on MARS.

We use different selection strategies to identify various local regions and conduct local attacks using both fixed textures and optimized textures. Then, we compare their performance across different detection networks for the same dataset and training network conditions. YOLOv3 is selected as the training network, whereas YOLOv3, YOLOv5s, YOLOv5x, YOLOv5m, YOLOv5n, and YOLOv5l are selected as the detection networks. All detection networks are trained with identical settings using the Carla dataset. Figure 6 and Figure 7 show the experimental results.

The first row, which is labeled “fixed texture,” uses a fixed camouflage for local attacks with different region selection strategies. The first column contains sample images of the original unmodified targets. We aim to mitigate the impact of the perturbation on the detector to highlight the effects of different region selection strategies on the attack effectiveness. The second row, which is labeled “optimized texture,” employs optimized patches for local attacks with different region selection strategies. The first column contains sample images of full-body attacks, which serves as the baseline. We aim to demonstrate the superiority of local attacks using the MARS strategy. The comparison strategies for region selection are as follows: The second and third columns show fixed regions that are manually selected based on expert guidance and highlight the edge and center regions, respectively. The fourth column shows the randomly selected regions. The fifth column shows critical decision regions that are selected by MARS. We evaluate the AP for these attack scenarios, and Table 1 shows the results.

**Performance Variations Between YOLOv3 and YOLOv5:** The differences in performance between YOLOv3 and YOLOv5 can be attributed to variations in their architectural complexity and depth. YOLOv5 models are generally deeper and incorporate more advanced features such as enhanced backbone networks and better anchor box strategies, which can influence their susceptibility to adversarial attacks. Specifically, YOLOv5l, being the largest variant, exhibits lower attack effectiveness due to its increased robustness and capacity to generalize from complex patterns, whereas smaller variants like YOLOv5s are more vulnerable due to their reduced complexity. These architectural differences explain why the attack performance varies across different YOLOv5 variants compared to YOLOv3.

Table 1 reveals significant differences between the Fixed(center) and Fixed(edge) strategies. Edge regions have lower visibility from different viewpoints than central regions, which leads to varied contributions during attacks. The AP after the Fixed(center) attacks is consistently lower than that after the Fixed(edge) attacks, which indicates that object edges are not equivalent to decision boundaries in neural networks. Random regions show inconsistent performance. With a fixed texture, the AP decreases to 0.967, which is better than the values of the fixed regions (0.980 and 0.978). However, with the optimized texture, the AP only decreases to 0.726, which is less effective than the values of the fixed regions (0.719 and 0.184). Thus, although random regions cover a broader area, their fragmented nature hampers the effective optimization and deception of neural networks. Additionally, YOLOv3 and YOLOv5 models respond differently to various attack strategies due to their distinct network architectures. YOLOv3 tends to be more sensitive to centralized attacks, whereas YOLOv5 models, especially larger variants, exhibit varied sensitivity based on their depth and feature extraction capabilities. Our proposed MARS strategy achieves AP decreases of 0.618–0.188 under optimized conditions, so AP surpasses all other strategies and closely approximates the full-body attack results.

Compared with the baseline, the attack efficiency (AE) for both fixed texture and optimized texture attacks is shown below, and the superior performance of the MARS strategy is highlighted.

Table 2 and Table 3 show that the AE of our proposed MARS method consistently outperforms the baseline and other local region selection strategies. The variations in AE across different YOLOv5 variants indicate that deeper and more complex networks like YOLOv5l are more resilient to adversarial attacks, requiring more concentrated and effective perturbations to achieve significant AE. In YOLOv3, MARS achieved an AE of 2.615, which is more than double the baseline value (0.911). In the YOLOv5 series, MARS achieved at least 0.608, which significantly outperformed the control group (0.532). The average AE for MARS was 1.7235, i.e., a 0.986 (134%) improvement over the baseline. These experiments demonstrate that, compared with other methods, our method effectively balances the coverage and region completeness and significantly improves stability and transferability. This result confirms the feasibility of using local adversarial attacks with the Maximum Aggregated Region Sparseness (MARS) strategy on 3D objects to attack detectors.

A significant reduction in AP signifies that the adversarial attack effectively diminishes the model’s capability to accurately detect and classify objects. In practical terms, this could lead to scenarios where critical objects, such as vehicles in autonomous driving systems, go undetected or are misclassified, potentially causing safety hazards. However, the extent of AP reduction must be carefully managed to avoid rendering the system non-functional, which could be impractical or easily noticed by human operators.

However, due to differences in network depth and width, the robustness to adversarial examples varies. In particular, the YOLOv5l network consistently shows lower attack effectiveness. This variation highlights the need for tailored adversarial strategies that consider the architectural nuances of different detection models. Addressing the network complexity and enhancing adversarial robustness will be a focus of future work.

### 4.3. Attack Transferability

This section of the experiment has two main objectives: to determine whether MARS can be combined with different texture modification methods for attacks and to assess the attack effectiveness of models that are trained on YOLO detectors and applied to other detectors.

We select the full region, Fixed(center) region, and MARS region, which perform well and are representative of previous experiments, as variables. Using FCA [13] and DAS [60] for texture optimization, we compare the resulting adversarial outcomes across different detection networks. To ensure the independence of the results, the detection networks are selected outside the YOLO series and include Mask R-CNN, Cascade R-CNN, Faster R-CNN, SSD, and RetinaNet.

As shown in Table 4, FCA and DAS exhibit varying performance across different region selection strategies. Full attacks consistently demonstrate stable results, where FCA and DAS achieve average AP decreases of 0.631 and 0.627, respectively. For the Fixed(center) region, Fixed(center) + FCA achieves an average AP decrease of 0.356, whereas Fixed(center) + DAS achieves an average AP decrease of 0.525. For the MARS region, MARS + FCA achieves an average AP decrease of 0.488, and MARS + DAS achieves an average AP decrease of 0.662. The MARS attack consistently outperforms other local attack methods, particularly with DAS, where it even outperforms the full attack. Thus, the critical decision regions identified by MARS align well with the decision boundaries of the model and demonstrate strong transferability with texture modification methods. Furthermore, the enhanced performance of MARS across different detection networks suggests that the aggregation and sparsity constraints effectively target universally critical regions, making the adversarial perturbations more versatile and robust against various model architectures. This adaptability is crucial for real-world applications where multiple detection systems may be in use.

Considering the attack effectiveness across different detection networks, the MARS attack outperforms other local attack methods on all networks. Specifically, MARS + DAS exceeds the performance of other local region selection strategies and surpasses the full attack in all networks except Cascade R-CNN. This superior performance is likely due to the MARS strategy’s ability to focus perturbations on regions that are consistently influential across different models, enhancing both the attack’s effectiveness and its transferability.

### 4.4. Attack Performance for Different Factors

In this section, we adjust various parameter coefficients to explore the significance of each loss parameter in this paper.

The training model is a YOLOv3 network trained for one epoch on the Carla dataset. Since we must only compare the effects of different coefficients, the selected detectors are also the YOLOv3 and YOLOv5s networks trained for one epoch on the Carla dataset.

First, we conduct experiments with different combinations of loss parameters. The adversarial loss, which ensures the fundamental effectiveness of the attack, is not adjusted. During training, the loss parameters are set as follows: $loss_{total}$ (all), $L_{adv}$ (single detection loss), $\alpha \cdot L_{agg}+\gamma \cdot L_{adv}$ (with aggregation regularization), and $\beta \cdot L_{sparse}+\gamma \cdot L_{adv}$ (with minimization regularization).

**Figure 8:**
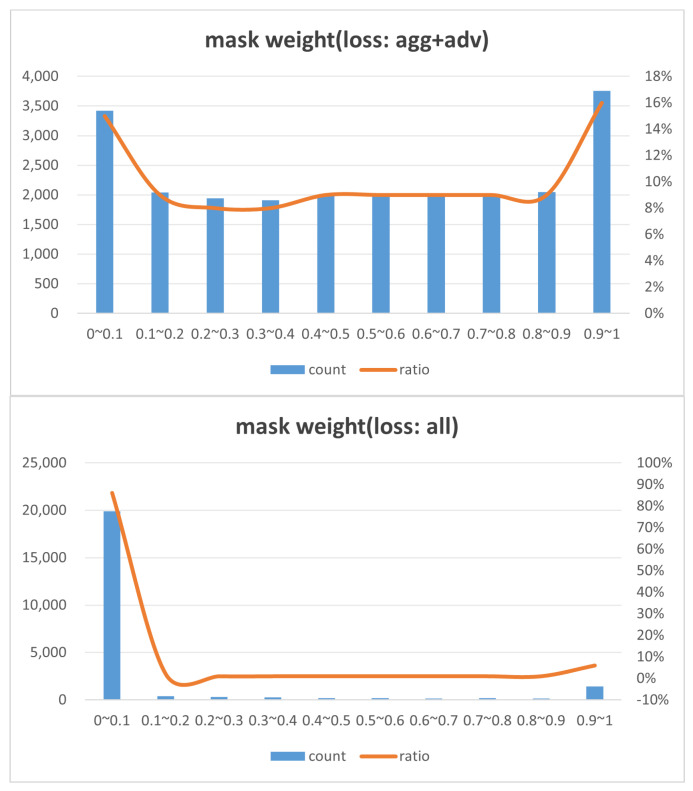
Mask weight distribution: (**top**) $loss=L_{agg}+L_{adv}$; (**bottom**) $loss=L_{agg}+L_{sparse}+L_{adv}$.

A comparison of Figure 8 shows that only pursuing aggregation during training tends to result in a uniform distribution of mask weights. The resulting regions lack constraints on the mask area and mask weight range and consequently do not possess decisive characteristics. Table 5 shows that with only the adversarial loss ($L_{adv}$) set, the AP decreases to 0.686 and 0.704. When only aggregation regularization ($L_{agg}$) is used, the AP only decreases to 0.77 and 0.655, which indicates poor attack performance. Conversely, when only sparseness regularization ($L_{sparse}$) is used, the AP decreases to 0.213 and 0.482, which significantly enhances the attack performance. This demonstrates that sparsity regularization is crucial for concentrating adversarial perturbations in critical regions, thereby increasing the attack’s effectiveness while maintaining stealthiness. The combined loss settings of $L_{agg}$ and $L_{sparse}$, which are ultimately adopted in this paper, achieve the best overall performance, identify universal decision regions, and successfully execute attacks.

In the second part of the experiment, we focus on adjusting the pre-parameters for the aggregation loss. As shown in Table 6, adjustments to parameter $L_{agg}$ reveal that increasing the parameter gradually improves the attack performance. However, after reaching a certain balance, the efficiency begins to decline. The mid-range values exhibit relatively stable results across all networks. This indicates that there is an optimal range for the aggregation coefficient where the aggregation of adversarial regions is maximized without causing over-concentration that could potentially dilute the attack’s effectiveness. The analysis indicates that the $L_{agg}$ coefficient controls the degree of aggregation in local regions. Increasing the coefficient yields more complete regions, which provides more possibilities for the optimization process. This behavior enables the generation of various continuous patterns that can deceive deep neural networks, which significantly impacts the attack effectiveness. Coefficient $L_{adv}$ controls the network attack effect; to better enhance the attack efficiency, it is essential to consider the balance among the loss coefficients. This experiment briefly explores the impact of the parameter coefficients on the experimental results. In future research, we will more precisely define the significance of coefficient $L_{agg}$ and delve deeper into the relationships among various coefficients.

In this part of the experiment, we keep other coefficients constant while adjusting the pre-parameter for the sparsity coefficient to modify the number of masks generated during optimization. This process enables us to compare the impact of the mask size on the attack efficiency. Table 7 shows the AP results. Since the variable here is the number of masks, we switch the metric to AE for easier comparison in Table 8.

As shown in Table 7, regarding the attack effectiveness, the number of masks has minimal effect when the count exceeds 2000. However, significant precision changes occur when the mask count fluctuates within the 0–2000 range. This result suggests that for this physical target, the optimal number of core critical regions detected by deep neural networks is less than 2000. This finding supports the main premise of this study: traditional adversarial attacks often optimize non-critical regions, which wastes computational resources. Our proposed method effectively identifies a sufficient number of local critical regions, reduces computational costs, enhances the visual effects, and achieves excellent attack performance across different detection networks under various mask counts.

As shown in Table 8, reducing the number of masks leads to a noticeable decrease in precision impact but a slight increase in attack efficiency. We observe that reducing the number of masks consistently increases the attack efficiency, which further emphasizes the importance of studying critical decision regions. When the mask count decreases during training, the computational speed improves, which is negligible in small-scale training but highly significant in large-scale large-model training. The aim of our study was to ensure rapid training and excellent visual effects while achieving significant attack effectiveness. The improvement in attack efficiency positively reflects this goal. Overall, our method consistently achieves robust adversarial results across different networks under most mask count settings, which demonstrates its broad applicability.

**Ethical Considerations:** The development and deployment of adversarial attacks pose significant ethical concerns, particularly regarding their potential misuse in critical systems such as autonomous driving, surveillance, and security infrastructures. These attacks can undermine the reliability and safety of systems that people depend on daily, leading to severe societal and economic consequences. To mitigate these risks, it is essential to implement robust defense mechanisms, promote responsible research practices, and establish regulatory frameworks that govern the use of adversarial technologies. Additionally, raising awareness about the vulnerabilities of machine learning models can encourage the development of more resilient systems and ethical guidelines for deploying such technologies.

**Societal Risks:** Adversarial attacks against critical systems such as autonomous vehicles, security surveillance, and infrastructure monitoring pose significant societal risks. These attacks can lead to accidents, breaches of privacy, and disruptions of essential services, potentially causing widespread harm. The ability to deceive detection models undermines trust in automated systems and can have far-reaching implications for public safety and security. To address these risks, it is imperative to develop robust defense mechanisms, enforce strict ethical guidelines, and promote collaboration between researchers, policymakers, and industry stakeholders to ensure that advancements in adversarial attacks do not compromise societal well-being.

## 5. Conclusions

In this study, we propose a novel method for identifying critical decision regions on the target surface and successfully achieving a balance between the visual effect and attack effectiveness in localized adversarial attacks. This approach demonstrates superior effectiveness compared to other region selection strategies and highlights its potential for targeted adversarial attacks. Moreover, when our region search strategy is combined with texture optimization, it yields outstanding attack performance and transferability. By focusing on smaller regions, this method reduces the introduction of non-transferable noise features and minimizes the likelihood of local optima in the attack model. Notably, in certain scenarios, our experimental results surpassed the effectiveness of the full-coverage optimization attack. Despite these strengths, a limitation of our method is the challenge of maintaining coherence between region optimization and subsequent texture optimization. Independently conducting these steps can introduce irrelevant noise, which may detract from the overall attack effectiveness. Future research will address this issue by investigating dual-parameter optimization techniques to simultaneously refine adversarial regions and textures. This integrated approach is expected to enhance the coherence and efficacy of adversarial attacks and advance the field of targeted adversarial strategies. Future avenues for improvement involve developing more sophisticated aggregation techniques, integrating MARS with other adversarial methods to create more potent attack strategies, and refining the approach to maintain effectiveness against increasingly robust detection systems. Addressing these challenges will further advance the capabilities of adversarial attacks, highlighting the necessity for ongoing research into model robustness and the development of more resilient detection frameworks.

## Figures and Tables

**Table 1:** AP of different region selection strategies.

Detector	Clean	Fixed Texture				Optimized Texture				
	Clean	Edge	Center	Random	Ours	Full	Edge	Center	Random	Ours
yolov3	0.983	0.98	0.978	0.967	0.899	0.071	0.719	0.184	0.726	0.174
yolov5m	0.991	0.99	0.99	0.989	0.931	0.123	0.935	0.745	0.912	0.373
yolov5n	0.988	0.985	0.985	0.969	0.888	0.306	0.956	0.787	0.887	0.523
yolov5l	0.953	0.952	0.952	0.944	0.831	0.421	0.907	0.815	0.879	0.765
yolov5s	0.964	0.962	0.956	0.955	0.902	0.289	0.919	0.567	0.805	0.337
yolov5x	0.994	0.994	0.993	0.972	0.815	0.235	0.842	0.639	0.853	0.502

**Table 2:** AE (fixed texture) of different region selection strategies.

Detector	Fixed (Edge)	Fixed (Center)	Random	Ours
mask_num	2000	2000	2000	2000
yolov3	0.01	0.016	0.052	**0.272**
yolov5m	0.003	0.003	0.006	**0.194**
yolov5n	0.01	0.01	0.061	**0.323**
yolov5l	0.003	0.003	0.029	**0.394**
yolov5s	0.006	0.026	**0.029**	0.2
yolov5x	0	0.003	0.071	**0.579**

**Table 3:** AE (optimized texture) of different region selection strategies.

Detector	Full	Fixed (Edge)	Fixed (Center)	Random
mask_num	6466	2000	2000	2000
yolov3	0.911	0.842	0.639	**0.75**
yolov5m	0.868	0.935	0.815	**0.842**
yolov5n	0.682	0.956	0.787	**0.639**
yolov5l	0.532	0.907	0.815	**0.765**
yolov5s	0.675	0.919	0.567	**0.514**
yolov5x	0.759	0.842	0.639	**0.502**

**Table 4:** AP (optimized texture) with different texture optimizers.

Method	Mask Pattern	Mask R-CNN	Cascade R-CNN	Faster R-CNN	SSD	RetinaNet	Average AE
	clean	0.764	0.723	0.716	0.705	0.751	**0.732**
FCA	Full	0.131	0.052	0.069	0.199	0.055	0.101^↓0.631^
	Fixed(center)	0.444	0.357	0.34	0.374	0.366	0.376^↓0.356^
	MARS	0.314	0.209	0.188	0.237	0.27	0.244^↓0.488^
DAS	Full	0.178	0.083	0.154	0.078	0.032	0.105^↓0.627^
	Fixed(center)	0.259	0.202	0.19	0.199	0.186	0.207^↓0.525^
	MARS	0.112	0.066	0.053	0.069	0.049	0.070^↓0.662^

**Table 5:** AP (different factor combinations).

Loss	agg+sparse+adv	agg+adv	sparse+adv	adv
yolov3	0.174	0.77	0.213	0.686
yolov5	0.337	0.655	0.482	0.704

**Table 6:** AP (different factors: agg_loss).

agg_loss	0.5	0.75	1	1.25	1.5	1.75	2
yolov3	0.2	0.167	0.174	0.173	0.174	0.183	0.172
yolov5	0.397	0.365	0.337	0.334	0.328	0.43	0.33

**Table 7:** AP (different factors: number of masks).

Mask Numbers	0	6466	5000	4000	3000	2000	1500	1000	500
yolov3	0.983	0.0719	0.129	0.142	0.148	0.174	0.216	0.299	0.597
yolov5	0.964	0.289	0.374	0.39	0.426	0.337	0.495	0.527	0.696

**Table 8:** AE (different factors: number of masks).

Mask Numbers	6466	5000	4000	3000	2000	1500	1000	500
yolov3	0.911	1.104	1.359	1.8	2.615	3.306	4.423	4.992
yolov5	0.675	0.763	0.928	1.16	2.027	2.022	2.826	3.466

## Data Availability

The data associated with this research are available online. The dataset is available at https://drive.google.com/drive/folders/1vspvRxnZ3shOV4kM5ELcO9-xztapBThS?usp=sharing (accessed on 20 June 2024).
